# Distinguishing Radiculopathies from Mononeuropathies

**DOI:** 10.3389/fneur.2016.00111

**Published:** 2016-07-13

**Authors:** Jennifer Robblee, Hans Katzberg

**Affiliations:** ^1^Division of Neurology, University Health Network (UHN), University of Toronto, Toronto, ON, Canada

**Keywords:** radiculopathy, focal neuropathy, mononeuropathy, neuromuscular, nerve root

## Abstract

Identifying “where is the lesion” is particularly important in the approach to the patient with focal dysfunction where a peripheral localization is suspected. This article outlines a methodical approach to the neuromuscular patient in distinguishing focal neuropathies versus radiculopathies, both of which are common presentations to the neurology clinic. This approach begins with evaluation of the sensory examination to determine whether there are irritative or negative sensory signs in a peripheral nerve or dermatomal distribution. This is followed by evaluation of deep tendon reflexes to evaluate if differential hyporeflexia can assist in the two localizations. Finally, identification of weak muscle groups unique to a nerve or myotomal pattern in the proximal and distal extremities can most reliably assist in a precise localization. The article concludes with an application of the described method to the common scenario of distinguishing radial neuropathy versus C7 radiculopathy in the setting of a wrist drop and provides additional examples for self-evaluation and reference.

## Introduction

Although nerve conduction studies (NCS) and electromyography (EMG) are standard tests in the evaluation of focal peripheral neuropathies (1), newer techniques, including peripheral nerve ultrasound and MRI neurography, have started to gain acceptance (2). Similarly, evaluation for radiculopathy often requires NCS/EMG as well as MRI of the cervical or lumbosacral spine, particularly in the case of sensory radiculopathy, which is not well evaluated with electrophysiology. Imaging and electrophysiology are not always readily accessible and, thus, a strong understanding of the clinical features that help distinguish these two very common peripheral neurological localizations is paramount.

This article focuses on an approach to distinguish two types of peripheral nerve lesions: monoradiculopathies and mononeuropathies. This exercise is a purely artificial scenario, which arises when one has excluded alternate localizations on the differential diagnoses, including the plexus and central localizations. The article will also not deal with the numerous etiologies that can affect the nerve root or peripheral nerve and have been eloquently summarized in other articles and textbooks (3).

The current article can be used by anyone with basic medical training, from medical students, residents, and fellows to neurologists, and other medical specialists encountering this common problem as well as other clinicians, such as neurology nurse practitioners and physiotherapists. The learning environment includes any clinical setting where patients with focal neuropathies will present, including the emergency room or primary care clinic as well as consultation opportunities, in the neurology or neuromuscular clinic. The specific learning objectives include the following: (1) to learn an approach that uses components of the basic neurological examination to distinguish common mononeuropathies from radiculopathies, (2) to review the distinguishing features between C7 radiculopathy and radial neuropathy, L5 radiculopathy and fibular neuropathy, L2–4 radiculopathy and femoral neuropathy, S1/2 radiculopathy and tibial neuropathy, C6 radiculopathy and median neuropathy, and C8 radiculopathy and median/ulnar neuropathy, (3) to realize the limitations of the presented approach.

Although the learners may have encountered similar approaches during the learning of the normal neurological examination (4–7), the rationale for learning the current method is that a systematic approach will help organize the appropriate clinical features helpful in differentiating localizations. The significance of learning this method well is that it can be applied to multiple scenarios and can form a base on which to add additional layers of neuromuscular localization, including differentiating between plexus lesions and the mononeuropathies and radiculopathies covered here. Multiple sources exist to reference the appropriate neuromuscular anatomy, and the learner should become intimately familiar with these in order to achieve success in the exercise (8, 9).

## Approach to the Distinguishing Mononeuropathies from Radiculopathies

### Pedagogical and Conceptual Framework

The framework on which the current approach is built on takes advantage of the unique properties between mononeuropathies and radiculopathies across the spectrum of the neurological examination. This includes differentially involved reflexes and components of the sensory examination, as well as uniquely involved muscles and muscle groups tested during the motor examination that can help distinguish localizations. When testing strength, the approach also uses the fact that proximal muscles are more often involved in radiculopathies than distal mononeuropathies. This concept allows the learner to organize key muscles and movements during the learning process and encourages a systematic approach to the localization exercise. The following is a comprehensive description of this method.

### Clinical Approach

Any patient newly presenting with neurologic deficits should have a full screening neurologic examination, with expansion of any relevant parts of the examination. In monoradiculopathies and mononeuropathies aspects of the examination, including mental status, coordination, and cranial nerves, are generally found to be intact, with a few exceptions, such as involvement of a Horner’s syndrome in the setting of a C8 radiculopathy. The approach to distinguishing monoradiculopathy versus mononeuropathy is outlined in Table 1 and described below.

**Table 1:** **Approach to distinguish monoradiculopathy from focal neuropathy (tabular format)**.

	Distal	Proximal	Reflexes	Sensory
Monoradiculopathy	What distal muscles are affected innervated by this nerve root, but not this nerve?	What proximal muscles are affected innervated by this nerve root, but not this nerve?	Is there a unique reflex, which is involved in dysfunction of this nerve root, but not the nerve?	Is there a dermatomal pattern sensory loss or irritative sign?
Focal neuropathy	What distal muscles are affected innervated by this nerve, but not this nerve root?	What proximal muscles are affected innervated by this nerve, but not this nerve root?	Is there a unique reflex, which is involved in dysfunction of this nerve, but not the nerve root?	Is there a nerve distribution sensory loss or irritative sign?

### Sensory Examination

Sensory abnormalities can be classified as “irritative” or “negative” not only on history but also using examination maneuvers. The sensory examination should include all the major modalities: light touch, pinprick, temperature sensation, vibration, and proprioception, however, when trying to map nerve or nerve root territories, testing light touch or pinprick is the most precise method of demarcating borders. The negative signs include areas of numbness (anesthesia/hypesthesia). Irritative signs use provocative maneuvers to irritate areas of nerve dysfunction in an attempt to localize the area of pathology.

#### Eliciting Irritative Signs

Irritative signs can be used to identify monoradiculopathies and include Spurling’s maneuver in the arms and the straight leg raise in the legs. To perform Spurling’s maneuver, the head is tilted toward the symptomatic arm with neck in extension. A positive test is radicular pain or paresthesias in the symptomatic arm (10). If negative, gentle downward loading of the head may be helpful. The straight leg raise is performed in the supine position, and the symptomatic leg is raised to above 45° with the knee in extension. A positive test is pain radiating past the knee. If there is no pain, the foot can be dorsiflexed to put further tension on the nerve root (11).

There are also irritative signs used to identify mononeuropathies, where any nerve with a segment that runs superficially in a vulnerable position can be tested as part of a Tinel’s test. The most common of these maneuvers is the Tinel’s maneuver at the wrist, which is described as percussing a nerve over the carpal tunnel to elicit numbness, paresthesia and/or pain within the distribution of the median nerve (12). The same maneuver can be performed on the ulnar as it traverses across the elbow in the ulnar groove or through the wrist in Guyon’s canal, although the diagnostic value has been questioned (13). The radial nerve can be tested at the spiral groove in the upper arm or as it exits the deep fascia of the forearm (14). In the legs, the fibular nerve can be percussed at the fibular head in suspected focal neuropathy there (15) and the lateral cutaneous nerve of the thigh can be percussed at the lateral hip in the setting of meralgia paraesthetica. In those same regions, the nerve can be palpated to rule out enlargement or nodularity of the nerve (16). Specific to the median nerve, another classic technique is Phalen’s maneuver. This is performed by asking the patient to press the dorsum of the hands against one another with wrists in the full flexion. This position should be held for up to a minute. The test is positive if it elicits or increases paresthesias in the median nerve territory.

#### Eliciting Negative Sensory Signs

The goal is to find a pattern of sensory deficits that fit either a dermatome or nerve distribution, acknowledging that there are many common areas of sensory disturbance between mononeuropathies and monoradiculopathy (17). For example, sensory disturbances in the lateral leg and dorsum of the foot are common to both L5 radiculopathy and fibular neuropathy and cannot be used to distinguish between these two localizations. Individual variability in nerve and nerve root territories can also make it difficult to precisely map out numbness in every individual. With these caveats, there occasionally are areas that are unique to the nerve or nerve root in question that can be helpful in diagnosis. This includes testing the thumb (C6) or digit 3 (C7) in distinguishing these localizations from radial neuropathy (mediodorsal aspect of the hand). Sensory “splitting” of digit 4 does not routinely occur in the setting of C7 (digit 3) or C8 (digits 4 and 5) radiculopathy, and can also help point toward a median or ulnar neuropathy. In the lower extremity, sensory loss in the medial leg is more consistent with a femoral neuropathy (distal saphenous branch) than L3 radiculopathy, which affects sensation most commonly in the thigh and over the knee only.

### Deep Tendon Reflexes

As with the sensory and motor examination, unique involvement of reflexes can be helpful in distinguishing mononeuropathies from monoradiculopathy (18). Reflexes that should be checked include the biceps (C5/C6, musculocutaneous), brachioradialis (C5/C6, radial), triceps (C6/7, radial), knee (L2/L3/L4, femoral), and ankle (S1, tibial). If the presenting complaint is in the hand, the finger jerk, a C8 reflex, can be added. Pectoral reflexes, a C5 reflex, can be added if there is proximal arm weakness. If the presenting complaint is in the leg, the medial hamstring reflexes can be added, especially if the localization differential includes L5 radiculopathy versus tibial or fibular neuropathy. When including reflexes outside the standard set, such as the finger jerks, pectoral reflex, or medial hamstring reflexes, one looks mainly for asymmetry. Absence of the reflex bilaterally is less helpful than ipsilateral absence of the reflex.

### Motor Examination

Motor examination should focus on identification of weakness, which is considered a negative motor symptom and usually very helpful in distinguishing peripheral localizations. Other findings, including atrophy and positive motor symptoms, such as fasciculations, often follow the same patterns as weak muscles and should be evaluated for, however, as less often localizing.

A careful screen of strength should be done using the Medical Research Council (MRC) scale (0 to 5) to grade the extent of weakness. Movements to include in the upper extremities should include the following: shoulder abduction (C5, axillary), elbow flexion (C5/C6, musculocutaneous), elbow extension (C6/C7, triceps), wrist extension (C6/C7, radial), finger extension (C7/C8, radial), wrist flexors and pronators (C6/7, median/ulnar), distal thumb flexors (C8, median), and finger abduction (C8/T1, ulnar). Extra muscles can be added to this set based on the specific localization(s) being considered. In the lower extremities, the following movements should at least be included: hip flexion (L1/L2, femoral), hip abduction (L5, superior gluteal), hip adduction (L2/L3/L4, obturator), knee extension (L2/L3/L4, femoral), knee flexion (L5/S1, sciatic), ankle dorsiflexion (L4/L5, deep fibular), ankle inversion (L5, tibial), toe extension (L5, deep fibular), and plantarflexion (S1/S2, tibial). As with the arms, extra muscles can be added to this set depending on nerve or nerve root being tested. The goal is to find a pattern of weakness in both proximal and distal muscles that is unique to either a radicular myotome or peripheral nerve distribution that can be used to distinguish the two lesions (19).

## Case Scenario: The Dropped Wrist – C7 Radiculopathy Versus Radial Neuropathy

A common presentation to the neurology clinic is wrist and finger extension weakness, which can have multiple localizations, including C7 radiculopathy and radial neuropathy. C7 radiculopathy is the most common radiculopathy in the cervical level as the C7-T1 vertebral segment is most mobile in the neck, vulnerable to degenerative disk disease and spinal stenosis. The long course of the radial nerve with points of compression at the spinal groove and Arcade of Frohse makes it a nerve vulnerable to compression. The following approach to the physical examination can help distinguish these two, once central causes and other neuromuscular localizations are excluded. The distinguishing features between C7 radiculopathy and radial neuropathy are outlined in Table 2.

**Table 2:** **Distinguishing examination features between C7 radiculopathy and radial neuropathy**.

	Distal	Proximal	Reflexes	Sensory
**C7 radiculopathy**	**Wrist pronators**	**Various shoulder movements (not useful due to multiple innervation)**	*Triceps reflex*	**Negative: third digit hypo/anesthesia**
	**Wrist flexors**			
	*Wrist extensors*	*Elbow extension*		**Irritative: Spurling’s maneuver**
	*Finger extensors*			
**Radial neuropathy**	**Flexion of brachioradialis**		**Brachioradialis reflex**	**Negative: first dorsal webspace hypo/anesthesia**
	*Wrist extensors*	*Elbow extension (if lesion is proximal to triceps branch)*	*Triceps reflex (if lesion is proximal to triceps branch)*	
	*Finger extensors*			

*The word highlighted in italics indicate commonalities between monoradiculopathy and focal neuropathy, and those in bold indicated the distinguishing examination findings*.

### Evaluating Sensation

C7 radiculopathies can cause sensory changes in the third digit. Radial neuropathies can cause sensory changes in the dorsum of the hand between the web of the thumb and index finger. Irritative maneuvers include Spurling’s maneuver and Tinel’s sign. Recall that Tinel’s sign can be performed in the upper arm or mid-forearm to support a radial mononeuropathy, while a positive Spurling’s maneuver would support a cervical radiculopathy.

### Deep Tendon Reflexes

As the triceps reflex can be impaired in both C7 radiculopathy and proximal radial neuropathy, it is not helpful in distinguishing between these two localizations. By contrast, impairment of the brachioradialis reflex can be helpful as it could indicate radial neuropathy over C7 radiculopathy when involved.

### Differential Muscle Strength Testing

First, ensure that the muscles that are innervated by both the C7 root and the radial nerve are affected to ensure that the primary differential diagnosis is between these two localizations. These include the triceps proximally and the wrist extensors of the forearm distally, the latter which also have C6 contribution. Next step is to find muscles that are only innervated by C7, and not by the radial nerve and vice-versa, considering these in the context of proximal versus distal muscles of the arm. Although there are proximal C7, non-radial muscles that help to move the shoulder and stabilize the scapula, such as latissimus dorsi and pectoralis major, these are not often involved in isolated C7 radiculopathy due to multiple innervation. Wrist flexors and pronators are such distal C7 innervated muscle groups innervated largely by the median and ulnar nerves, not by the radial nerve.

The other half of this step is identifying muscles innervated by the radial nerve that are not C7 innervated. Again there are no proximal muscles in these categories, but there is one major distal muscle. As stated in the reflexes, brachioradialis is a radial C6 (not C7) innervated muscle distally which can help to diagnose a radial neuropathy versus C7 radiculopathy if it is weak. To complete the motor assessment, look for atrophy or fasciculations in the muscles of the arm mentioned above, comparing to the non-symptomatic arm if needed.

## Conclusion

An approach to narrowing down a focal neuropathy versus monoradiculopathy localization includes first identifying areas of sensory loss, which are distinct, and signs of focal nerve irritation using maneuvers, such as Tinel’s signs or nerve root irritation with maneuvers, such as straight leg raise or Spurling’s maneuver can help further distinguish the two localizations. Reflexes can be differentially affected in nerve and nerve root lesions and clinicians should consider less commonly tested reflexes, including the medial hamstring L5, finger flexor C8, and pectoral C5 reflexes. Finally, the clinician should evaluate for differential patterns of weakness that exist between the two localizations in (a) proximal muscles, which are more often involved in radiculopathies, and (b) distal muscles, where there is usually at least one muscle or muscle group, which can help distinguish the lesions.

Limitations of using the current method include the fact that it is an artificial exercise in localizing peripheral lesions. The clinician should remember to consider other localizations, including central brain and spine disorders, as well as other disorders of the peripheral neuroaxis, including the lumbosacral and brachial plexus. In addition, the current method is a purely clinical method used to localize lesions, which can sometimes be unreliable in patients with comorbid musculoskeletal conditions, other pathologies limiting the examination or in situations with poor patient cooperation, such as unconscious patients. As such, clinician should be aware that NCS/EMG and imaging may also be used to confirm suspected clinical localization and that full neurological and medical evaluation for the underlying cause of focal neuropathy and monoradiculopathy should follow this exercise. Finally, the learner should realize that this exercise is a purely localization exercise, and etiologies causing the final pathology in question should still be reviewed.

It is also important to note that this approach still requires the learner to review peripheral nerve anatomy and that additional approaches, including use of clinically distinguishing features between mononeuropathies and radiculopathies not presented here, could also be used to localize these peripheral lesions. In the authors’ experience that applying this methodology can be a powerful tool in learning an approach to patients with neuromuscular conditions; however, it is important for the learner to continue to review the scenarios presented as well as clinical neuroanatomy if learning is to be consolidated. This approach should also form a basic framework upon which to develop an approach to distinguishing more complex localizations, including the brachial and lumbosacral plexus and other mononeuropathies.

In spite of these limitations, the current approach uses the common language of the neurological examination universally taught in medical curricula applied to the specific problem of identifying mononeuropathies versus radiculopathies. The approach can be used by a variety of clinicians to accurately localize these common neuromuscular processes in a structured and organized manner. Tables 2–5 provide additional common focal neuropathy versus radiculopathy scenarios encountered in clinical practice and the ways to distinguish these localizations that can be used during the learning process by self-testing. By continuing to review these scenarios, the learner will gain the structured approach used by neuromuscular clinicians when encountering these types of patients and consultations.

**Table 3:** **C6 radiculopathy versus median neuropathy**.

L5 Radiculopathy versus fibular neuropathy				
	Distal	Proximal	Reflexes	Sensory
**L5 radiculopathy**	**Ankle inversion**	**Hip abduction**	**Medial hamstring reflex**	**Negative: portion of the plantar surface of the foot and strip along lateral leg and thigh**
	*Ankle/toe dorsiflexion*			*Negative: dorsal surface of the foot* + *lateral leg*
				**Irritative: straight leg raise test**
**Fibular neuropathy**	*Ankle/toe dorsiflexion*	–	–	*Negative: superficial fibular branch: dorsal foot and lateral leg*
				*Negative: deep fibular branch: first dorsal webspance*

*The word highlighted in italics indicate commonalities between monoradiculopathy and focal neuropathy, and those in bold indicated the distinguishing examination findings*.

**Table 4:** **L2/L3/L4 radiculopathy versus femoral neuropathy**.

L2/L3/L4 Radiculopathy versus femoral neuropathy				
	Distal	Proximal	Reflexes	Sensory
**L2/L3/L4 radiculopathy**	**Ankle dorsiflexion (may be spared as also innervated by L5 myotome)**	**Hip adduction**	*Knee reflex*	**Medial upper thigh**
		*Knee extensors*		
				±**lateral thigh (L2)**
				**+***medial knee and lower thigh (L3)*
				+*medial lower leg (L4)*
**Femoral neuropathy**	–	**Hip flexion**	*Knee reflex*	*Medial knee and lower thigh* + medial lower leg
		*Knee extension*		

*The words highlighted in italics indicate commonalities between monoradiculopathy and focal neuropathy, and those in bold indicated the distinguishing examination findings*.

**Table 5:** **S1/S2 radiculopathy versus tibial neuropathy**.

	Distal	Proximal	Reflexes	Sensory
**S1/S2 radiculopathy**	*Soleus/gastrocnemius*	**Hip extension and knee flexion**	*Ankle*	*Lateral/inferior foot* + posterior thigh
**Tibial neuropathy**	*Soleus/gastrocnemius*	-	*Ankle*	*Lateral/inferior foot* + posterior thigh

## Author Contributions

Both authors conceived the article and drafted and reviewed all aspects of the manuscript.

## Conflict of Interest Statement

The authors declare that the research was conducted in the absence of any commercial or financial relationships that could be construed as a potential conflict of interest.
